# Effects of AMCOP^®^ Elastodontic Devices on Skeletal Divergence and Airway Dimensions in Growing Patients

**DOI:** 10.3390/jcm14155297

**Published:** 2025-07-27

**Authors:** Gianna Dipalma, Alessio Danilo Inchingolo, Filippo Cardarelli, Antonio Di Lorenzo, Fabio Viapiano, Laura Ferrante, Francesco Inchingolo, Daniela Di Venere, Andrea Palermo, Grazia Marinelli, Angelo Michele Inchingolo

**Affiliations:** 1 Department of Interdisciplinary Medicine, School of Medicine, University of Bari “Aldo Moro”, 70124 Bari, Italy; giannadipalma@tiscali.it (G.D.); alessiodanilo.inchingolo@uniba.it (A.D.I.); drfilippocardarelli@libero.it (F.C.); antoniodilorenzo95@gmail.com (A.D.L.); viapianofabio96@gmail.com (F.V.); lauraferrante79@virgilio.it (L.F.); daniela.divenere@uniba.it (D.D.V.); grazia.marinelli@uniba.it (G.M.); angeloinchingolo@gmail.com (A.M.I.); 2 Department of Experimental Medicine, University of Salento, 73100 Lecce, Italy; andrea.palermo@unisalento.it

**Keywords:** elastodontic therapy, functional appliances, skeletal divergence, vertical growth control, airway space, oropharyngeal dimensions, interceptive therapy, craniofacial development, orthodontic treatment

## Abstract

**Objectives**: This study aimed to evaluate the effects of AMCOP^®^ elastodontic appliances on cephalometric parameters of skeletal divergence and upper airway dimensions in growing patients, comparing treated individuals with an untreated control group. **Methods**: A total of 60 subjects (30 treated with AMCOP^®^ devices and 30 controls) were selected, with mean ages of 8.67 ± 1.3 and 9.19 ± 0.8 years, respectively. The AMCOP^®^ appliances, designed for mixed dentition, were worn for 1 h during the day and throughout the night for 6–8 months. Cephalometric analyses were conducted at the beginning (T0) and end (T1) of treatment. Statistical analyses were performed using multivariable linear regression models to assess changes in skeletal and airway parameters, with significance set at *p* < 0.05. **Results**: Significant reductions were observed in Ans-Snp^Go-Gn (*p* = 0.0351), SN^Go-Gn (*p* = 0.0091), and FMA (*p* < 0.001) in the treated group compared to controls, indicating improved mandibular rotation. Upper airway spaces (SPAS, MAS, IAS) increased significantly, suggesting enhanced airway patency. Regression models confirmed the positive impact of AMCOP^®^ therapy on skeletal and airway outcomes, particularly in subjects with pronounced vertical discrepancies. **Conclusions**: AMCOP^®^ elastodontic devices effectively promote anterior mandibular rotation and reduce mandibular plane inclination in hyperdivergent patients, contributing to balanced craniofacial growth. The expansion of pharyngeal spaces indicates potential respiratory benefits. Future research is needed to confirm long-term stability and address variability in treatment response.

## 1. Introduction

Skeletal divergence is a critical aspect of orthodontic analysis, affecting not only craniofacial growth but also airway morphology with implications for respiratory function. Skeletal divergence is a critical element in orthodontic diagnosis and treatment planning, as it affects not only craniofacial development but also upper airway morphology and, consequently, respiratory function [1,2,3,4,5]. In orthodontic patients, it is essential to understand the differences between normodivergence, hypodivergence, and hyperdivergence in order to plan effective treatments [6,7,8]. Normodivergence is characterized by a balance between the vertical and anteroposterior growth components, resulting in a harmonious profile and a balanced distribution of masticatory forces [9,10,11,12].

The typical cephalometric parameters of these patients, with an FMA between 20° and 25° and an SN^GoGn angle around 32–35°, reflect ideal occlusal stability and balanced growth [13,14,15,16,17]. Hypodivergence, on the other hand, is manifested by a predominant anteroposterior growth and a less inclined mandibular plane, associated with a more closed gonadal angle [18,19,20]. This cephalometric configuration, with an FMA of less than 20° and an SN^GoGn of less than 30°, suggests a mandible that tends to rotate anteriorly, accentuating a prognathic profile [21,22,23,24].

Hyperdivergence is one of the most complex phenotypes to treat, as it is characterized by predominantly vertical growth [25,26,27,28]. Cephalometrically, FMA values greater than 25° and an SN^GoGn angle often greater than 37° are observed, indicating posterior rotation of the mandible, resulting in a marked inclination of the mandibular plane [29,30,31,32]. This skeletal pattern is not only aesthetically unfavorable but also functionally problematic, as posterior mandibular rotation can constrict the upper airway and impair both breathing and posture. Previous studies have documented this airway compromise in hyperdivergent patients, emphasizing the need for treatment modalities that support anterior mandibular rotation.

This growth pattern results in increased anterior facial height and a convex facial profile associated with decreased masticatory muscle strength [33,34,35]. Posterior rotation of the mandible has not only aesthetic but also functional implications, as it can reduce the upper airway space, affecting breathing and posture [36,37].

In hyperdivergent subjects, reduced pharyngeal space has been observed to predispose to less efficient breathing, increasing the importance of strategies to promote anterior rotation of the jaw [38,39,40,41]. The management of verticality requires complex therapeutic approaches that combine the use of orthodontic, orthopedic and, in some cases, surgical devices to correct skeletal and dentoalveolar discrepancies [42,43].

In this context, elastodontic devices—particularly multifunctional cranio-occlusal–postural (AMCOP^®^) bioactivators—have emerged as an innovative, non-invasive approach [44,45]. These devices are thermo-activatable and are designed to harmonize the skeletal and dentoalveolar bases without the need for advanced constructions, thus avoiding invasive maneuvers. They act as “elastodontic spaces” where teeth position themselves under the guidance of neuromuscular forces without rigid constraints, promoting long-term occlusal stability [46,47,48,49,50]. In growing patients, these devices offer the advantage of promoting greater occlusal stability without invasive treatment, potentially improving airway patency and supporting balanced craniofacial growth [51,52,53]. Importantly, few studies have systematically addressed how anterior mandibular rotation may influence airway space by altering the morphology and position of key craniofacial structures, including the soft palate, tongue, genioglossus, and geniohyoid muscles. Given these gaps in current knowledge, this study aims to evaluate the effects of AMCOP^®^ elastodontic appliances.

With particular attention to the effects on cephalometric values characteristic of skeletal divergence and changes in upper airway dimensions, we compared a group of treated patients with an untreated control group. Although several studies have investigated the relationship between mandibular rotation and facial aesthetics, few have systematically explored how anterior mandibular rotation in growing patients may influence upper airway morphology—particularly in relation to soft tissue structures such as the soft palate, tongue posture, and associated muscle dynamics (e.g., genioglossus and geniohyoid muscles). These interactions are particularly relevant in hyperdivergent subjects, where vertical skeletal discrepancies are often associated with reduced airway patency and compromised breathing function.

Given these gaps in current knowledge, this study aims to evaluate the effects of AMCOP^®^ elastodontic appliances on both cephalometric parameters of skeletal divergence and upper airway dimensions in growing patients. The objective is to assess whether such non-invasive elastodontic therapy can modulate craniofacial development and improve airway space, thereby supporting both functional and orthopedic outcomes. Understanding these effects may have clinical implications for early interceptive treatment strategies in pediatric orthodontics.

## 2. Materials and Methods

### 2.1. Sample Selection

The study received approval from the Ethical Committee of the Policlinico of Bari, and informed consent was obtained from the parents of each patient. A study sample of 30 subjects (13 males and 17 females, mean age 8.67 ± 1.3 years) treated with AMCOP^®^ bioactivators was collected from patients referred to the Department of Orthodontics at the Policlinico of Bari and to an orthodontic practice.

The treated group was compared with an untreated control group of 30 subjects (16 males and 14 females, mean age 9.19 ± 0.8 years) selected from the archives of the American Association of Orthodontists Foundation Craniofacial Growth Legacy Collection (http://www.aaoflegacycollection.org, Bolton Brush Growth Study, Burlington Growth Study, Iowa Growth Study, Mathews Growth Study, Michigan Growth Study, and Oregon Growth Study, accessed on 1 April 2024).

The control group was subjected to the same inclusion and exclusion criteria as the treatment group. The inclusion criteria were as follows:

- Patients who had undergone lateral cephalometric X-rays both before and after treatment.
- Patients with mixed dentition.
- Prepubertal or pubertal stage in skeletal growth at initial observation.

Exclusion criteria were as follows:

- Previous orthodontic treatment.
- History of adenoidectomy or tonsillectomy.
- Craniofacial syndromes and abnormalities.

Each patient provided informed consent. Documentation for the orthodontic treatment plane included extraoral and intraoral pictures, orthodontic study models, panoramic radiographs, and latero-lateral teleradiographs taken at the beginning and completion of treatment/period of observation (T0).

### 2.2. Treatment Protocol

A thermo-activatable elastic device with functional, orthopedic, joint, neuromuscular, occlusal, and postural action, the Multifunctional Cranio-Occlusal-Postural Harmonizer (Armonizzatori Multifunzionali Cranio-Occluso-Posturali, AMCOP^®^) bioactivator, was used as part of the treatment strategy.

The vestibular and lingual flanges constituting the AMCOP^®^ bioactivators delineate a central free space that represents the equilibrium zone between the forces exerted by the tongue and those generated by the cheeks and lips, allowing the teeth to position themselves naturally. The elevated vestibular flanges function as a lip bumper by displacing the perioral muscles and proprioceptively stimulating the bone matrix.

The AMCOP^®^ bioactivators simultaneously engage both dental arches, exerting an orthopedic influence on the maxillary bones across transverse, sagittal, vertical, and torsional planes. The incorporation of a lingual ramp and tongue button guides the tongue toward the palate, facilitating the re-establishment of correct lingual posture and function. Median incisions are strategically designed to avoid interference with the labial and lingual frenula as well as the retroincisal papilla. Additionally, two concavities in the canine draft regions are incorporated to prevent any interference with the canines [54,55,56].

The devices are composed of a polymer–elastomer blend available in two Shore hardness grades, 51 and 60, selected according to specific operational requirements. The material exhibits high elasticity, thermoplastic properties, and heat-activated adaptability, allowing it to conform to various arch forms. Additionally, the device can be expanded by immersion in hot water at approximately 70 °C for 30 s, followed by cooling in cold water to stabilize its modified shape [47,51]. AMCOP^®^ bioactivators are available in various sizes and colors, which correspond to different skeletal classes and arch forms. They are specifically designed to accommodate deciduous, mixed, and permanent dentitions, making them suitable for patients of all age groups.

In this study, patients used AMCOP^®^ designed for mixed dentition:

- First class devices.
- Second-class devices.
- Third-class devices.
- OPEN devices.

**Figure 1:**
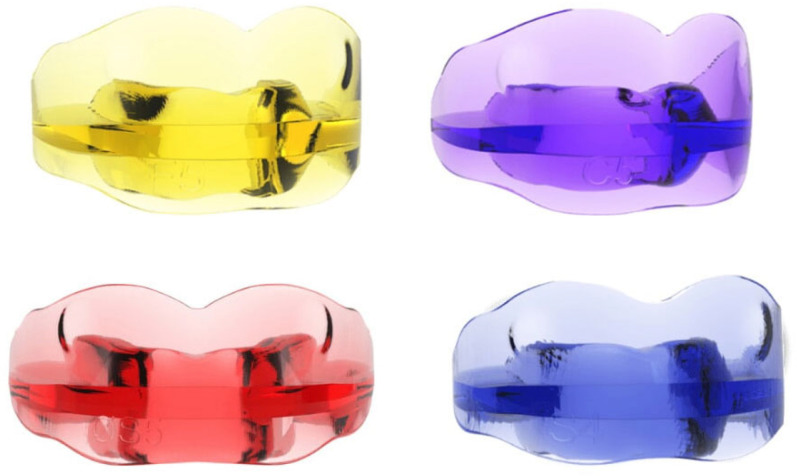
AMCOP^®^ I Class appliance.

Class I appliances (Class I skeletal pattern), indicated for basal skeletal disharmonies in the transverse and vertical planes, in the absence of a second- or third-class sagittal component, are made with four different arch shapes in relation to the type of cranial conformation of the patient and two different occlusal planes (arch shapes: F—suitable for dolichocephalic subjects with ogival arches and narrow palate; S—suitable for mesocephalic subjects with oval arches; OS—suitable for mesocephalic subjects with square arches; C—suitable for brachycephalic subjects with wide, rounded arches and flat palate. Chewing planes: INTEGRAL—flat occlusal plane, suitable for subjects with a normal bite; BASIC—thicker occlusal plane in the anterior region to increase the vertical dimension in subjects with a deep bite) (Figure 1).

**Figure 2:**
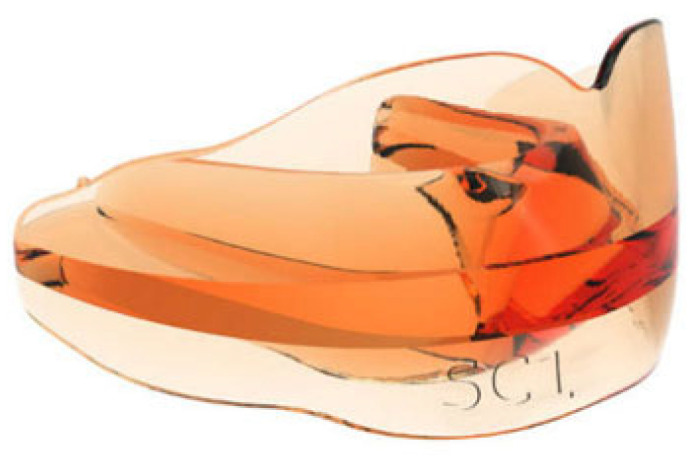
AMCOP^®^ II Class appliance (SC).

Class II (SC) appliances (Class II skeletal pattern) are used to correct mandibular retrognathia in Class II dysmorphia (Figure 2). Equipped with a mandibular anterior gliding plane, they control the growth of the upper jaw and increase the vertical dimension (in the case of a deep bite), correct the overjet by retroinclining the maxillary incisors and proinclining the mandibular incisors, and allow the improvement of temporomandibular joint dysfunction, labial incompetence, and facial aesthetics. Unlike other traditional functional devices such as the Twin-Block or the Bionator, the SC bioactivator does not require a construction bite.

**Figure 3:**
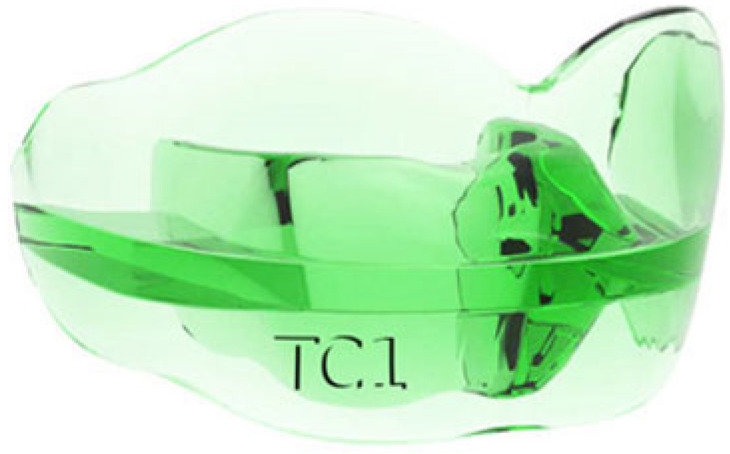
AMCOP^®^ Class III (TC) appliance.

Class III (TC) devices (Class III skeletal pattern) are indicated for the treatment of mandibular pseudoprognathia in Class III dysmorphia (Figure 3). The third-class AMCOP^®^ bioactivator is characterized by an occlusal plane that is inclined in a postero-anterior direction from top to bottom on the upper surface and in the opposite direction from bottom to top on the lower surface. This creates a pair of forces in the sagittal direction that acts as a brake on the mandibular growth.

**Figure 4:**
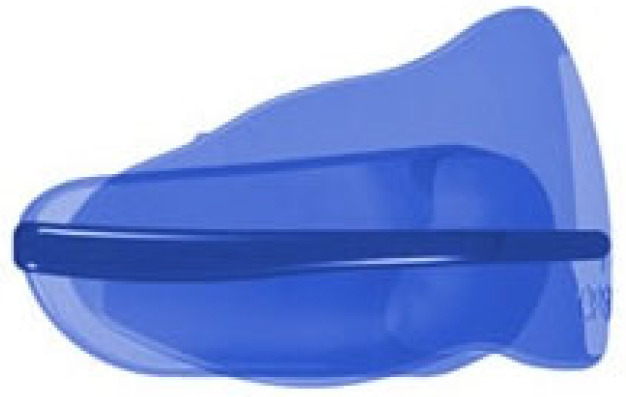
AMCOP^®^ OPEN appliance.

**Figure 5:**
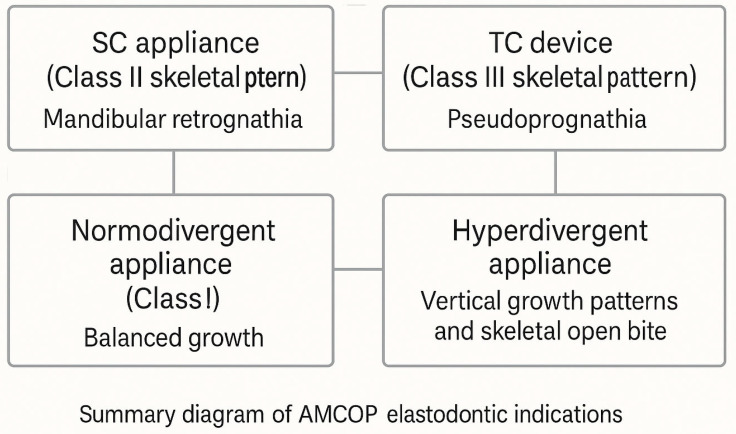
Summary diagram of AMCOP^®^ elastodontic devices and clinical indications.

OPEN appliances (skeletal open bite pattern) characterized by a posteriorly raised occlusal plane, are indicated in cases of skeletal open bite (Figure 4). Each device is produced in multiple sizes, with the sizing determined by measuring the transverse distance between the vestibular cusps of the upper first molars (Figure 5). Patients were instructed to wear the device for one hour during the day and throughout the night for a period of 6 to 8 months, after which it was used exclusively at night.

The follow-up period was standardized to a minimum of 6 months. Although some patients continued treatment for up to 8 months, all cephalometric analyses and comparisons were based on a fixed observation interval of 6 months from baseline (T0). Any minor variation in duration beyond this period was not statistically significant and was not found to affect the outcomes in regression analyses.

### 2.3. Cephalometric Analysis

Cephalometric studies were conducted for each patient at the beginning of the treatment/observation period (T0) and at the conclusion of therapy (T1). The DeltaDent^®^ 2.5.3 software was used for all cephalometric evaluations. Cephalometric dentoskeletal parameters and radiographic parameters related to airway dimensions were taken into account and then collected in a Microsoft Excel^®^ spreadsheet (version 16.88) and subjected to statistical analysis. All the values obtained are presented in Table 1.

### 2.4. Stastistical Analysis

The study’s outcomes were the reductions in Ans-Pns^Go-Gn, S-N^Go-Gn, and FMA and the increase in OVB in the control and study group. Secondary outcomes were represented by the increase in SPAS, MAS, and IAS in the same groups. Quantitative variables were described as means ± standard deviation (SD). Qualitative variables were described as percentages (proportion).

The mean reduction in Ans-Pns^Go-Gn, S-N^Go-Gn, and FMA and the mean increase in SPAS were compared between the study group and the control group via the Mann–Whitney non-parametrical test due to a non-normal distribution. The OVB, MAS, and IAS mean increases were compared between the two groups via the t-Student test, as their distributions were found to be normal.

A multivariable linear regression model was then fitted for each outcome, using it as the dependent variable. The subject’s group, sex, age at T0, and initial value of the specific parameter were used as independent variables. Finally, univariable regression models were fitted using only independent variables for which a significant association with our outcomes could be identified. For all statistical purposes, a *p*-value < 0.05 was considered an indicator of statistical significance.

The study database was built on Microsoft Excel^®^, version 16.88. All statistical analyses were conducted via StataMP17^®^. All data were treated in full respect of the European General Data Protection Regulation.

### 2.5. Sample Size Justification

A priori power analysis was conducted using G*Power software (version 3.1.9.7) to determine the minimum sample size required to detect a significant difference in cephalometric and airway parameters between the treated and control groups. Assuming a medium effect size (Cohen’s d = 0.65), an alpha level of 0.05, and a power of 0.80, the required sample size was calculated to be 27 subjects per group. Therefore, the inclusion of 30 participants per group was deemed sufficient to ensure adequate statistical power for hypothesis testing.

## 3. Results

**Figure 6:**
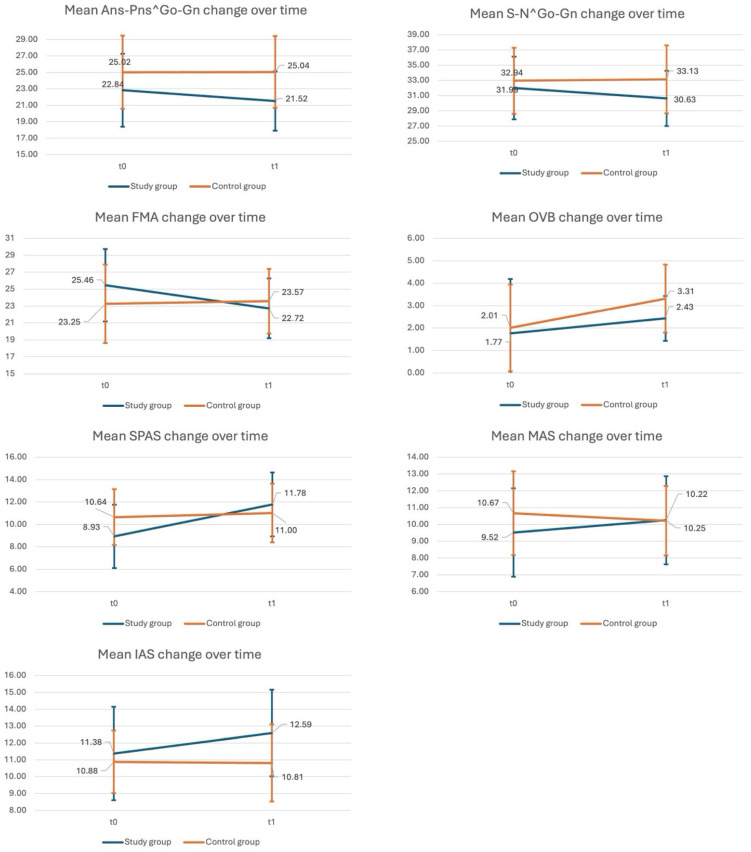
Changes of each parameter from T0 to T1.

Sixty subjects were enrolled, thirty for each group. A full set of information regarding the population can be found in Table 2 and Table 3. The changes of each parameter from T0 to T1 are represented in the graphs in Figure 6.

The mean Ans-Snp^Go-Gn reduction was significantly different between the study and control groups (z: −2.11; *p*-value: 0.0351). A significant difference between the two groups was also identified for the mean S-N^Go-Gn reduction (z: −2.61; *p*-value: 0.0091) and FMA reduction (z: −3.76; *p*-value < 0.001), respectively. The OVB increase, however, was not significantly different between the two groups (t: 1.11; *p*-value: 0.2719).

Regarding the secondary outcomes, the mean SPAS increase was significantly different between the study and control group (z: −3.47; *p*-value < 0.001). The MAS increase was also significantly different between the two groups (t: −2.11; *p*-value: 0.039), while the IAS increase was only significant as a one-way analysis, when tested for a greater increase in the study group (t: −1.97; *p*−value: 0.027).

### 3.1. Regression Analysis: Ans-Pns^Go-Gn Reduction

The multivariable linear regression model identified a significant positive influence of the experimental therapy on the reduction of Ans-Pns^Go-Gn (coeff.: 1.88; 95% CI: 0.71–3.04; *p*-value: 0.002). At the same time, greater initial values of Ans-Pns^Go-Gn determined a significantly greater reduction of this parameter over time (coeff.: 0.24; 95% CI: 0.12–0.37; *p*-value < 0.001). The patient’s sex and age did not impact significantly on Ans-Pns^Go-Gn reduction (*p*-value > 0.05).

The univariable regression confirmed a significant impact of the experimental therapy of the parameter reduction (coeff.: 1.33; 95% CI: 0.11–2.55; *p*-value: 0.033). Further, greater t0 values of Ans-Pns^Go-Gn were still proven to be significantly associated with a greater reduction (coeff.: 0.19; 95% CI: 0.05–0.32; *p*-value: 0.007).

### 3.2. Regression Analysis: S-N^Go-Gn Reduction

The multivariable regression showed a significantly positive effect of the study therapy on S-N^Go-Gn reduction (coeff.: 2.15; 95% CI: 0.85–3.46; *p*-value: 0.002). As per the previous parameter, the value of S-N^Go-Gn at t0 was also associated with greater reduction of this parameter over time (coeff.: 0.25; 95% CI: 0.10–0.41; *p*-value: 0.001). In addition to this, male sex was also related to better results of the therapy (coeff.: 1.49; 95% CI: 0.22–2.76; *p*-value: 0.023). Age at t0 was not significantly associated with the S-N^Go-Gn reduction (*p*-value > 0.05).

Univariate regression confirmed a positive influence on the S-N^Go-Gn reduction of both the study group (coeff.: 1.56; 95% CI: 0.19–2.93; *p*-value: 0.026) and t0 S-N^Go-Gn value (coeff.: 0.21; 95% CI: 0.05–0.37; *p*-value: 0.011). However, male sex did not show a significant impact on the S-N^Go-Gn reduction when analyzed singularly (*p*-value: 0.200).

### 3.3. Regression Analysis: FMA Reduction

Multivariable linear regression highlighted a significant impact of the experimental therapy on FMA reduction (coeff.: 2.12; 95% CI: 0.74–3.50; *p*-value: 0.003). At the same time, greater values of FMA at t0 were associated with greater reductions (coeff.: 0.38; 95% CI: 0.22–0.54; *p*-value < 0.001). Neither sex nor age at t0 were significantly associated with the FMA reduction (*p*-value > 0.05).

Univariable analysis confirmed the significantly higher decrease in FMA for both the experimental group (coeff.: 3.05; 95% CI: 1.50–4.60; *p*-value < 0.001) and greater initial values of FMA (coeff.: 0.44; 95% CI: 0.29–0.60; *p*-value < 0.001).

### 3.4. Regression Analysis: OVB Increase

The multivariable regression model showed a significant negative impact of the study intervention on the increase in OVB (coeff.: −0.92; 95% CI: −1.60–0.23; *p*-value: 0.009). The value of OVB at t0 was also negatively associated with increases in OVB (coeff. −0.83; 95% CI: −0.99–−0.68; *p*-value < 0.001), showing that subjects with a lower initial value have greater increases over time. Sex and age at the start of the treatment were not significantly associated with the OVB increase (*p*-value > 0.05).

When the experimental treatment was investigated in a univariable regression model, it showed no significant association with the OVB increase over time (*p*-value: 0.272). The initial OVB value, on the contrary, retained its significant negative association with the increase in the parameter (coeff.: −0.84; 95% CI: −0.99–−0.68; *p*-value <0.001).

### 3.5. Regression Analysis: SPAS Increase

The multivariable regression highlighted a significant positive association with the experimental treatment (coeff.: 1.92; 95% CI: 0.62–3.22; *p*-value: 0.004) and a significant negative association with the value of SPAS at t0 (coeff.: −0.43; 95% CI: −0.66–−0.20; *p*-value < 0.001). On the other hand, sex and age at t0 were not associated with SPAS’s increase over time (*p*-value > 0.05).

Univariable regression confirmed both observations, with the study group maintaining a positive influence (coeff.: 2.49; 95% CI: 1.17–3.80; *p*-value < 0.001) and the initial value of the parameter showing a negative impact on its increase (coeff.: −0.52; 95% CI: −0.75–−0.30; *p*-value < 0.001).

### 3.6. Regression Analysis: MAS Increase

When studied in a multivariable regression model, the MAS increase over time was only significantly associated with the baseline value of the parameter. In particular, a negative association was identified (coeff.: −0.43; 95% CI: −0.63–−0.23; *p*-value < 0.001). No other variable was significantly associated with MAS’s increase (*p*-value > 0.05). The significant influence of the starting value of MAS was then confirmed via univariable regression (coeff.: −0.46; 95% CI: −0.65–0.27; *p*-value < 0.001).

### 3.7. Regression Analysis: IAS Increase

Multivariable regression showed a significant influence of both the study intervention (coeff.: 1.56; 95% CI: 0.35–2.76; *p*-value: 0.012) and the starting value of IAS (coeff.: −0.52; 95% CI: −0.78–−0.27; *p*-value < 0.001) on its increase over time. Age at the beginning of therapy and sex were not significantly impactful (*p*-value > 0.05). However, when studied via univariable regression, only IAS’s value at t0 was confirmed to significantly impact the parameter’s increase (coeff.: −0.50; 95% CI: −0.76–−0.25; *p*-value < 0.001).

### 3.8. Summary of Multiple Linear Regression Models

To provide a clearer understanding of the relative contribution of each predictor variable to the outcome, standardized and unstandardized coefficients from the multiple linear regression models were calculated and are summarized in the following tables. Each model included group (treatment vs. control), sex, age at T0, and baseline value of the dependent variable. Adjusted R^2^ values and ANOVA results are also reported for model quality assessment (Table 4 and Table 5).

## 4. Discussion

Analysis of the results of this study showed that the AMCOP^®^ elastodontic appliances can produce significant improvements in cephalometric parameters of skeletal divergence and upper airway dimension, confirming the effectiveness of a non-invasive approach based on neuromuscular stimulation [57]. In the sample of growing patients, significant reductions in the Ans-Snp^Go-Gn, SN^Go-Gn, and FMA angles were observed, suggesting a positive influence on mandibular rotation and a more harmonious skeletal growth [46,58,59].

The reduction in the Ans-Snp^Go-Gn angle was particularly notable, indicating an improvement in skeletal divergence through an important orthopedic effect. This result supports the hypothesis that AMCOP^®^ bioactivators can promote anterior rotation of the mandible, reduce the inclination of the mandibular plane, and improve the aesthetics of the facial profile. The observed positive correlation between angle reduction and treatment efficacy highlights the potential of AMCOP^®^ devices to modulate growth in a three-dimensional manner, especially in patients with more pronounced vertical discrepancies [60,61,62].

The significant reduction in the SN^Go-Gn angle, which describes the inclination of the mandibular plane relative to the anterior skull base, has important implications for muscle function and mandibular biomechanics [63,64,65,66]. A more aligned mandible allows for an optimal distribution of chewing forces, with a reduction in muscle strain and an overall improvement in stomatognathic function [67,68,69,70].

The data suggests that the effectiveness of the treatment is particularly pronounced in subjects with initially more pronounced vertical discrepancies. The decrease in FMA is also an outcome of importance, as this parameter is a key indicator of vertical growth [71,72,73,74]. An increased FMA in hyperdivergent patients is associated with increased anterior facial length and a tilted mandibular plane [75,76,77,78].

The AMCOP^®^ device, by positively influencing this parameter, allows for better mandibular growth management and promotes a more harmonious profile. The effectiveness of functional devices in growing patients, as reported in the literature, exploits the adaptive potential of skeletal structures [79,80,81,82].

Regarding the upper airway, the analysis showed a significant increase in the SPAS, MAS, and IAS. The widening of the upper airway space (SPAS) suggests an improvement in airway patency, which is particularly relevant as posterior rotation of the mandible in hyperdivergent patients can reduce the pharyngeal space [83,84,85,86]. Treatment with AMCOP^®^ devices appears to counteract this effect by improving respiratory function.

The increase in the MAS between the soft palate and the posterior wall of the oropharynx is significant in patients with mandibular retrognathia, where a reduction in this space can affect breathing [87,88,89]. By promoting correct lingual posture and rehabilitation of the perioral musculature, AMCOP^®^ devices help to increase the MAS, suggesting a respiratory benefit [90,91,92]. The IAS, which is the distance between the base of the tongue and the back wall of the pharynx, also increased, indicating an improvement in lingual posture and more efficient breathing [55,93]. Statistical analysis confirmed that treatment with AMCOP^®^ devices was associated with significant cephalometric changes and airway expansion. Regressions highlighted the importance of considering variables such as baseline parameter values and patient gender to optimize outcomes. The relationship between mandibular rotation and airway morphology is likely mediated by several craniofacial factors. Anterior mandibular rotation, as promoted by AMCOP^®^ devices, may contribute to airway enlargement not only by repositioning the mandible forward but also by stretching and repositioning the genioglossus and geniohyoid muscles, which are functionally connected to the base of the tongue and the hyoid bone. This muscular traction can increase the oropharyngeal airway space by shifting the tongue anteriorly and downward [94,95,96]. Furthermore, mandibular advancement can indirectly influence the morphology and positioning of the soft palate, reducing its posterior displacement and increasing the dimensions of both the MAS and SPAS. Therefore, the airway improvement observed in treated patients may result from a combination of skeletal changes and functional adaptations involving both soft tissue structures and muscular dynamics [97,98,99]. Despite individual variability in treatment response and the need for further long-term studies, the data suggests a significant therapeutic potential for elastodontic devices in growing patients. Nevertheless, some limitations should be acknowledged. The present study lacks randomization and a control group, which may limit the strength of causal inferences. In addition, the absence of long-term follow-up data prevents the assessment of the stability and durability of the observed changes over time. Individual variability in treatment response, influenced by growth patterns and compliance, also represents a potential confounding factor. These limitations highlight the need for future prospective, controlled studies with extended follow-up periods to validate and expand upon the current findings.

## 5. Conclusions

This study aimed to evaluate the effects of AMCOP^®^ elastodontic appliances on skeletal divergence and upper airway dimensions in growing patients. The results demonstrated that AMCOP^®^ devices are effective in promoting anterior mandibular rotation and reducing vertical skeletal discrepancies, as evidenced by significant reductions in the Ans-Snp^Go-Gn, SN^Go-Gn, and FMA angles. These changes suggest a favorable influence on craniofacial growth and facial profile balance, particularly in hyperdivergent subjects.

Furthermore, a significant increase in upper airway dimensions (SPAS, MAS, IAS) was observed in the treated group, indicating a potential functional improvement in respiratory patency. These findings support the hypothesis that elastodontic therapy can modulate both skeletal and airway development through neuromuscular and orthopedic mechanisms.

However, limitations such as the lack of randomization and long-term follow-up must be acknowledged. Future controlled and longitudinal studies are needed to confirm the stability of these results and better understand the variability in patient response.

## 6. Patents

Title: dispositivo ortodontico-elastico-armonizzatore dento cranio facciale, scope: Italian, granted under n. 102015000057082.

Title: dispositivo ortodontico-elastico-armonizzatore dento cranio facciale, scope: International, granted under n. WO 2017/056010.

## Figures and Tables

**Table 1:** Radiographic parameters.

Parameters	Definitions	Mean Values (SD)
SNA	Angle between sella–nasion and nasion–point A segments.	82° (±2°)
SNB	Angle between sella–nasion and nasion–point B segments.	80° (±2°)
ANB	Angle between point A–nasion and nasion–point B segments.	2° (±2°)
Ans-Pns^Go-Gn	Intermaxillary angle, between bispinal plane (Ans-Pns) and mandibular plane (Go-Gn).	28° (±2°)
SN^Go-Gn	Mandibular angle between sella–nasion plane (S-N) and mandibular plane.	32° (±5°)
FMA	Angle between Frankfurt plane and mandibular plane.	25° (±3°)
OVJ (mm)	Overjet, distance on the sagittal plane between the upper and lower incisors.	2.5 (±2.5 mm)
OVB (mm)	Overbite, distance on the vertical plane between the upper and lower incisors.	2.5 (±2.5 mm)
SPAS	Superior posterior airway space, the distance along a line parallel to the gonion–menton plane (Go–Me) between the soft palate and the posterior wall of the nasopharynx.	n.d.
MAS	Middle airway space, the distance measured along a line parallel to the Go-Me plane between the posterior wall of the oropharynx and the lower tip of the soft palate.	n.d.
IAS	Inferior airway space, the distance between the posterior wall of the pharynx and the base of the tongue along the Go-Me plane.	n.d.

**Table 2:** Qualitative variables of the population.

**Qualitative Variables**		**N.**	**Percentage**
**Sex**	Male	29	48.33%
	Female	31	51.67%

**Table 3:** Quantitative variables of the population.

Quantitative Variables	CASE				CONTROL			
	T0		T1		T0		T1	
	Mean	SD	Mean	SD	Mean	SD	Mean	SD
Age (years)	8.67	±1.30	15.93	±0.83	9.17	±0.79	15.70	±0.99
SNA	83.30	±2.84	82.15	±2.47	81.15	±3.13	81.50	±2.99
SNB	77.13	±2.62	78.67	±2.11	81.50	±2.99	75.26	±3.01
ANB	5.95	±1.82	3.57	±1.18	5.94	±1.87	5.25	±2.59
Ans-Pns^Go-Gn	22.95	±4.36	21.50	±3.54	24.80	±4.37	24.90	±4.29
SN^Go-Gn	31.95	±4.06	30.20	±3.56	33.55	±4.26	33.85	±4.39
FMA	24.75	±4.22	22.75	±3.48	24.15	±4.54	24.40	±3.75
OVJ (mm)	4.95	±1.56	3.20	±0.99	5.65	±2.13	5.60	±2.24
OVB (mm)	1.90	±2.38	2.65	±0.98	1.90	±1.90	3.25	±1.49
SPAS	8.65	±2.78	12.25	±2.80	10.60	±2.45	10.70	±2.58
MAS	9.30	±2.59	9.45	±2.57	10.95	±2.45	10.45	±2.03
IAS	10.85	±2.72	12.40	±2.52	11.00	±1.82	10.35	±2.24

**Table 4:** Multiple regression model predicting FMA reduction.

Predictor	Unstd. B	Std. β	SE	95%CI for B	*p*-Value
Group (Treated = 1)	−2.12	−0.38	0.69	[−3.50, −0.74]	0.003
Sex (Male = 1)	0.14	0.03	0.59	[−1.04, 1.33]	0.791
Age at T0	−0.06	−0.02	0.35	[−0.76, 0.64]	0.869
FMA at T0	0.38	0.67	0.08	[0.22, 0.54]	<0.001

Model Summary: R^2^ = 0.57, Adjusted R^2^ = 0.53, Standard Error = 1.82. ANOVA: F (4, 55) = 18.12, *p* < 0.001.

**Table 5:** Multiple regression model predicting SPAS increase.

Predictor	Unstd. B	Std. β	SE	95%CI for B	*p*-Value
Group (Treated = 1)	1.92	0.34	0.63	[0.62, 3.22]	0.004
Sex (Male = 1)	−0.11	−0.02	0.56	[−1.24, 1.02]	0.848
Age at T0	0.03	0.01	0.31	[−0.59, 0.65]	0.919
SPAS at T0	−0.43	−0.55	0.11	[−0.66, −0.20]	<0.001

Model Summary: R^2^ = 0.48, Adjusted R^2^ = 0.44, Standard Error = 2.01. ANOVA: F (4, 55) = 12.75, *p* < 0.001.

## Data Availability

The original contributions presented in this study are included in the article. Further inquiries can be directed to the corresponding author.

## Abbreviations

The following abbreviations are used in this manuscript:

AMCOP^®^	Armonizzatori Multifunzionali Cranio-Occluso-Posturali
ANB	Point A–Nasion–Point B Angle
CI	Confidence Interval
FMA	Frankfurt Mandibular Plane Angle
GN	Gnathion
GO	Gonion
IAS	Inferior Airway Space
MAS	Middle Airway Space
OVB	Overbite
OVJ	Overjet
SD	Standard Deviation
SN	Sella–Nasion
SNA	Sella–Nasion–Point A Angle
SNB	Sella–Nasion–Point B Angle
SPAS	Superior Posterior Airway Space
T0	Initial Timepoint (Inizio del Trattamento)
T1	Final Timepoint (Fine del Trattamento)
